# Viral and immune profiles during the first wave of SARS-CoV-2 infection in hospitalized patients in Sardinia, Italy

**DOI:** 10.1038/s41598-025-90324-5

**Published:** 2025-02-24

**Authors:** Giulietta Venturi, Alessandra Gallinaro, Claudia Fortuna, Maria Franca Pirillo, Arianna Scoglio, Beatrice Di Carlo, Giulia Marsili, Zuleika Michelini, Antonello Amendola, Alberto Carocci, Stefania Dispinseri, Martina Borghi, Andrea Canitano, Chiara Falce, Alice Zappitelli, Gabriella Scarlatti, Maria Luisa Lixi, Alessandra Aste, Laura Masala, Silvia Baroncelli, Andrea Cara, Donatella Negri

**Affiliations:** 1 Department of Infectious Diseases, Istituto Superiore di Sanità, Rome, Italy; 2 National Center for Global Health, Istituto Superiore di Sanità, Rome, Italy; 3 National Center for the Control and Evaluation of Medicines, Istituto Superiore di Sanità, Rome, Italy; 4 Viral Evolution and Transmission Unit, IRCCS Ospedale San Raffaele, Milan, Italy; 5 Center for Gender-Specific Medicine, Istituto Superiore di Sanità, Rome, Italy; 6 Laboratory Medicine, Santissima Trinità Hospital, Cagliari, Italy

**Keywords:** SARS-CoV-2, Mucosal antibodies, Neutralizing antibodies, Sardinia, Immunology, Infectious diseases, Viral infection

## Abstract

We performed a retrospective immunological analysis of the antibody response in serum and in nasopharyngeal swabs (NPS) obtained from 46 individuals infected with ancestral SARS-CoV-2 Wuhan-Hu-1 strain during the first COVID-19 wave in Cagliari (Sardinia, Italy), with a 4-month follow-up after the hospital admission. We implemented a comprehensive antibody response in serum and in mucosal samples using assays established in our laboratories. In NPS we evaluated the viral load by real time PCR, presence and kinetics of anti-Spike IgG and IgA by ELISA as well as their anti-Wuhan neutralization activity, showing induction and persistence of anti-viral immunity at the mucosal level. Neutralizing antibodies were measured in serum and NPS using a safe pseudovirus-based assay validated after comparison with a standard neutralization test using live SARS-CoV-2. We evaluated cross-neutralizing antibodies against all the major early variants of concerns (VoC) in sera. Of note, we detected a remarkable reduction of neutralizing activity against BA.1 compared to BA.2 and BA.5 Omicron subvariants, which was confirmed in sera from an analogous cohort of patients at the San Raffaele hospital in Milan, a geographically distant region of Italy, infected with the ancestral virus during the same period of time.

## Introduction

Severe acute respiratory syndrome coronavirus 2 (SARS-CoV-2) is the betacoronavirus responsible for the coronavirus disease 2019 (COVID-19) pandemic. The COVID-19 pandemic started as a severe pneumonia outbreak from Wuhan city (Hubei Province, China) in December 2019 to quickly spread to other regions of Asia and soon after throughout the world, including Italy, which was the earliest country afflicted by the pandemic in Europe. COVID-19 pandemic affected severely Italy, especially early during the pandemic, with the majority of cases occurring in clusters within the northern administrative regions (i.e. Lombardy, Piedmont, Veneto and Emilia Romagna), whereas the Center-South area was marginally concerned by the pandemic, also thanks to the effective containment measures (lockdown) which were put in place to avoid viral spread, starting from March 9th 2020. Within the Center-South area, the island of Sardinia was among the regions with the lowest SARS-CoV-2 incidence rate, with 136.3 cases each 100,000 inhabitants at the end of August 2020 towards the onset of the second wave^1,2^. Sardinia’s insularity and geographical positioning (distance from mainland Italy) coupled with the lockdown measures may explain the low COVID-19 incidence rate.

Growing evidence suggests that a wide range of genetic variants and environmental factors^3^ affect the pathogenic fate of COVID‐19. The Sardinian population has a unique genetic heritage, including the human leukocyte antigens (HLA) diversity/haplotypes that impacted on virus susceptibility and disease severity^4–7^and despite the emergence of different SARS-COV-2 variants, the Sardinian population continues to maintain the lowest incidence of mortality and one of the lower rate of Intensive Care Unit (ICU) admission compared to other Italian regions^8^. To our knowledge, studies on the comprehensive characterization of functional humoral responses against SARS-CoV-2 during the first COVID-19 wave in Sardinia are missing. In addition, there are only few studies on the evaluation of cross-neutralizing activity against later variant of concerns (VoC) in patients infected with ancestral SARS-CoV-2 and before the vaccination campaign^9,10^.

This retrospective study aims at evaluating the dynamics of antibody responses to SARS-CoV-2 in serum and mucosal samples of Sardinian patients infected with ancestral SARS-CoV-2 Wuhan-Hu-1 strain and hospitalized during the first wave of COVID-19 (March–April 2020). Additionally, to investigate the degree of neutralizing activity against VoC of Sardinian patients, the neutralizing profile against early Omicron subvariants was compared to that obtained in sera from an analogous cohort of patients hospitalized during the same period at the San Raffaele Hospital in Milan (Lombardy) using the same pseudovirus assay developed in our laboratory^11^.

## Methods

### Study population

The study included 46 patients (≥ 18 years old) from the Santissima (SS) Trinità hospital in Cagliari (Sardinia, Italy) with confirmed SARS-CoV-2 infection based on positive real-time reverse-transcriptase polymerase chain reaction (RT-PCR) from a nasal-pharyngeal swab (NPS) and/or symptoms and radiological findings suggestive of COVID-19 pneumonia. All patients included in this study were admitted to the hospital between March and April 2020 with indicators of severe disease, according to the World Health Organization (WHO) guidelines^12^, and enrolled in the observational study “COVID-IMM: Analisi della risposta IMMunologica nei sieri di pazienti COVID-19”, reviewed and approved by the institutional Review Board (RB) (protocol number 260/2020/CE).

During the hospital recovery, routine blood tests included complete blood count with differential, renal and liver function tests, C-reactive protein (CRP) and lactate dehydrogenase (LDH), serum ferritin, and D-dimer. For each patient aliquots of sera and NPS were collected at different time points, stored at -80 °C, and sent for further analyses to the Istituto Superiore di Sanità (ISS). The patient samples were stratified according to the collection days from the day of hospitalization.

### Analysis of anti-SARS-CoV-2 IgG and IgM in serum

Anti-SARS-CoV-2 IgM and IgG antibodies targeting the Nucleoprotein and the Spike protein were measured in undiluted sera at the Cagliari’s hospital laboratory by chemiluminescence immunoassay (CLIA), using the iFlash-SARS-CoV-2 commercial kits and the iFlash analyzer, following the manufacturer’s instructions (YHLO Biotech Co. Ltd., Shenzen, China). Results are expressed in arbitrary units (AU)/ml with 10 AU/ml as a positive cut-off of the assays.

### Analysis of anti-Spike SARS-CoV-2 IgA and IgG in nasopharyngeal swabs

Nasopharingeal specimens were collected at the Hospital using the swabs (FLOQSwabs) placed into sterile tubes containing transport medium (UTM Universal Transport Medium, Copan, Italy) and frozen at − 80 °C until use. Samples were then thawed, inactivated at 56 °C for 30 min, centrifuged at 2800 × g for 10 min and the supernatants were aliquoted and stored at − 80 °C. Anti-Spike IgG and IgA in Nasopharyngeal swabs (NPS) were evaluated by ELISA using SARS-CoV-2 purified trimeric Spike^13^ as coating protein. Briefly, 96-well plates (Greiner bio-one, Frickenhausen, Germany) were coated with 0.2 µg/well of Spike protein overnight at 4 °C. After washing and blocking for 2 h with 200 µL of PBS containing 1% BSA (Sigma Chemicals), two fold serial dilutions of NPS starting from 1:10 were added to wells in duplicate and incubated for 2 h at room temperature. The plates were washed and biotin-conjugated goat anti-human IgG or IgA (Southern Biotech, Birmingham, AL, USA) was added to the wells for 2 h at room temperature. The plates were washed again before the addition of horse radish peroxidase (HRP)-conjugated streptavidin (AnaSpec, Fremont, CA, USA) for 30 min at room temperature. The antigen–antibody reaction was measured by using the 3.3,5.5-tetramethylbenzidine substrate (SurModics BioFX, Edina, MN, USA) and the reaction was stopped with 50 µL of 1 M H_2_SO_4_. Endpoint titers were determined as the reciprocal of the highest dilution giving an absorbance value at least equal to threefold that of background (NPS from healthy individuals). To set up the ELISA, different positive controls were used, including total Ig isolated from the plasma of a COVID-19 convalescent patient, anti-Spike monoclonal IgA (the Native Antigen Company, Kidlington, UK) and IgG (COVA2-15^13^) antibodies.

Total IgG and IgA in NPS were evaluated by sandwich ELISA using Bethyl Laboratories reagents (Montgomery, TX, USA). Briefly, plates were coated with polyclonal goat anti-human IgG or IgA antibodies, and serial dilutions of an internal standard (purified human serum IgG or purified human IgA) and two fold serial dilutions of NPS, starting from 1:100, were added to wells in duplicate and incubated for 2 h at room temperature. The next steps were the same as described above. The concentration of Ig in the NPS was calculated using an interpolation curve by GraphPad Prism (GraphPad Software Inc., San Diego CA, USA). Since the concentration of total IgG and IgA is different in each individual, to compare antigen-specific antibody isotype IgG and IgA in the NPS, we normalized the data expressing the results as % of Spike-specific Ig/total Ig, for each antibody isotype.

### SARS-CoV-2 neutralization assays

All sera were tested for the presence of neutralizing antibodies (nAbs) directed towards the ancestral virus-derived Spike, using a pseudovirus-based assay developed in our laboratory, as previously described^11,14,15^. The assay was validated by comparing the nAb titer in serum of COVID-19 infected patients (N = 19) with a neutralization assay using live SARS-CoV-2 as described below. To ensure comparability between the two assays, statistical correlation tests were run.

#### Live virus-based neutralization assay

The virus neutralization test (VNT) was performed using the SARS-CoV-2 isolate (GenBank: MT066156.1; Cat: NR-52284; SARS-Related Coronavirus 2, Isolate Italy-INMI1; BEI Resources, Manassas, VA, USA). The virus propagation and the neutralization assay were performed as already described^14^. Briefly, after heat-inactivation for 30 min at 56 °C, serum samples, starting from 1:10 dilution, were mixed with an equal volume of 50 TCID50 SARS-CoV-2 viral solution and incubated for 1 h at 37 °C and 5% CO2 in a humidified atmosphere. A 100 µL of virus–serum mixture was subsequently added to a 96-well plate containing a 70% confluent Vero E6 cell monolayer (Cercopithecus aethiops derived epithelial kidney, ATCC C1008). After 4 days of incubation at 37 °C and 5% CO2 in a humidified atmosphere, the plates were inspected by an inverted optical microscope for the presence/absence of cytopathic effect (CPE). The highest serum dilution that protected more than the 50% of cells from CPE was taken as the neutralization titre. The VNT titer was expressed as the reciprocal of the highest serum dilution showing protection from viral infection and CPE.

#### Pseudotyped virus-based neutralization assay

Lentiviral vector-based pseudoviruses expressing luciferase (LV-Luc) pseudotyped with Spike (LV-Luc/Spike) were obtained by transient transfection of 293 T Lenti-X cells on 10 cm Petri dishes (Corning Incorporated-Life Sciences, Oneonta, NY, USA), using the pGAE-Luc lentiviral transfer vector plasmid expressing the luciferase coding sequence, the packaging plasmid pAd-SIV3 + and each of the pseudotyping plasmid expressing Spike proteins, as previously described^11,14^. Forty-eight hours post transfection, the supernatants containing the LV-Luc pseudoviruses were collected, filtered with a 0.45 µm pore size filter (Millipore Corporation, Billerica, MA, USA) and titered in Vero E6 cells seeded in a 96-well plate Viewplate (PerkinElmer, Groningen, Netherlands) at a density of 2.2 × 10^4^ cells/well. After 48 h, luciferase expression was determined by the Britelite plus Reporter Gene Assay System (PerkinElmer) and measured in a Varioskan luminometer (Thermo Fisher Scientific, Waltham, MA, USA). Dilutions providing 2 × 10^5^ relative luminescence units (RLU) were used in the neutralization assay. Briefly, sample serial twofold dilutions starting from 1:40 or 1:80 for serum and 1:10 for NPS were incubated in duplicate with the LV-Luc/Spike for 30 min at 37 °C in 96-deep well plates (Resnova, Roma, Italy), and thereafter added to Vero E6 cells seeded in a 96-well Isoplate (PerkinElmer) at a density of 2.2 × 10^4^ cells/well. Virus-only and cell-only controls were included. After 48 h, luciferase expression was determined by the Britelite plus Reporter Gene Assay System. RLU data points were converted to a percentage neutralization value, calculated relative to virus-only controls. Results are expressed as inhibitory concentration (ID) 90, 75 and 50 corresponding to the sample dilution giving 90%, 75% and 50% inhibition of infection, respectively compared to the virus-only control wells. ID values were calculated with a linear interpolation method^11,14^.

### Neutralizing activity against SARS-CoV-2 variants

To determine the cross neutralizing activity against variants of concerns (VoC), different LV-Luc/Spike pseudoviruses were generated by replacing the Wuhan-Hu-1 Spike with Spikes derived from several VoC, including Beta, Delta and Omicron (BA.1, BA.2, BA.4/5), using the same methodology summarized above and previously described^14^. The pseudoviruses were used to compare the cross-neutralizing activity against Omicron subvariants in selected Sardian patients and in a cohort of COVID-19 convalescent patients from IRCCS San Raffaele Hospital in Milan, a geographically distant Italian population. Serum samples were obtained from patients (matched for age, sex ratio, and anti-Wuhan neutralization titers) enrolled in a previous study (COVID-19 Patients characterization, Biobank, Treatment response and Outcome Predictor [COVID-BioB], EC protocol number 34/int/2020, clinicalTrials.gov NCT04318366)^16^.

### Quantification of SARS-CoV-2 RNA in NPS

The viral RNA from NPS were extracted using the DSP Virus/Pathogen midi kit with automated QIAsymphony (Qiagen). Viral copies were evaluate by Real Time PCR as indicated by the protocol from the U.S. Centers for Disease Control and Prevention (CDC)^17^. Briefly, the real time PCR was carried out on CFX 96 Biorad with a mixture reaction of 20 µl containing 5 µl of extracted RNA, 5 µl of 4 × TaqPath™ 1-Step RT-qPCR Master Mix (Thermo Fisher Scientific Inc.), and primers and probes for the N2 gene; the human ribonuclease P gene (RP constitutive gene) was amplified as an internal control. SARS-CoV-2 titer, expressed as TCD50/ml equivalent (TCD50_eq_/ml), was assessed by crossing point Ct values compared with a standard curve obtained from ten-fold serial dilutions of a titrated virus stock (SARS CoV2 Italy INMI #52,284 (III) with concentration 7.5 × 10^6^ TCD50/ml.

### Statistical analysis

Data were analyzed with GraphPad Prism 9.5.1 (GraphPad Software Inc.) and were expressed as the mean ± standard error of the mean (SEM) or median with 95% CI. The correlation was evaluated by Spearman correlation analysis, the comparison by the Wilcoxon matched-pairs signed rank test and the mixed-effects analysis with multiple comparisons. *P* values < 0.05 were used as the threshold for statistical significance. Descriptive statistics for the demographic characteristics of the study group were expressed as median (95% CI) for continuous variables, and as percentage for the categorical variables.

## Results

### Study cohort characteristics

Forty-six patients were enrolled in this retrospective observational study. Patients’ characteristics are reported in Table 1; at the hospital admission the median age was 62.5 years (95% CI 58–72), most of them male (67.4%). A relevant fraction had one or more co-morbidity (86.6%), with hypertension (46.6%) and obesity (20.0%) being the most frequent. At admission, symptoms consisted prevalently of fever, fatigue, and respiratory difficulties often associated with dyspnea. The median hospitalization duration was 50 days (95% CI 33–59) . Overall six patients died (13.0%), 16 patients (34.8%) were admitted to the Intensive Care Unit (ICU) of whom 4 died after ICU entry (25.0%). The set of laboratory blood tests recorded according to SS Trinità Hospital protocol is shown in Supplementary Table S1. Serum samples and nasopharyngeal swabs (NPS) were collected at hospital admission and at later time points and were used at the hospital for clinical purposes. Samples still available (not all the time points and not for all patients) were then used for the analyses performed in the context of this study.

**Table 1:** Characteristics of the SS Trinità COVID-19 study population (N = 46).

Characteristics	N (%)	Missing data
Age, years (median, 95%CI)	62.5 (58–72)	0
Sex Male	31 (67.4%)	0
Ethnicity		0
Caucasian	46 (100%)	
Co-morbidities		1
Hypertension	21 (46.6%)	
Obesity	9 (20.0%)	
Coronary Artery Diseases (CAD)	6 (13.3%)	
Rheumatoid arthritis	4 (8.9%)	
Cancer	4 (8.9%)	
Neurodegenerative disease (ND)	3 (6.7%)	
Number of co-morbidities		1
None	6 (13.3%)	
1	16 (35.5%)	
2	12 (26.7%)	
3 or more	11 (24.4%)	
Symptoms at disease onset		1
General (fever, headache, fatigue/malaise, myalgia)	38 (84.4%)	
Respiratory (cough, dyspnea)	36 (80.0%)	
Gastrointestinal (diarrhea, vomiting/nausea)	8 (17.8%)	
Median days of hospital stay (95%CI)	50 (33–59)	0
Median days of ICU stay of 16 patients (95%CI)	14 (7–38)	0

ICU: intensive care unit.

### Analysis of anti-SARS-CoV-2 humoral responses in patients’ serum

**Fig. 1:**
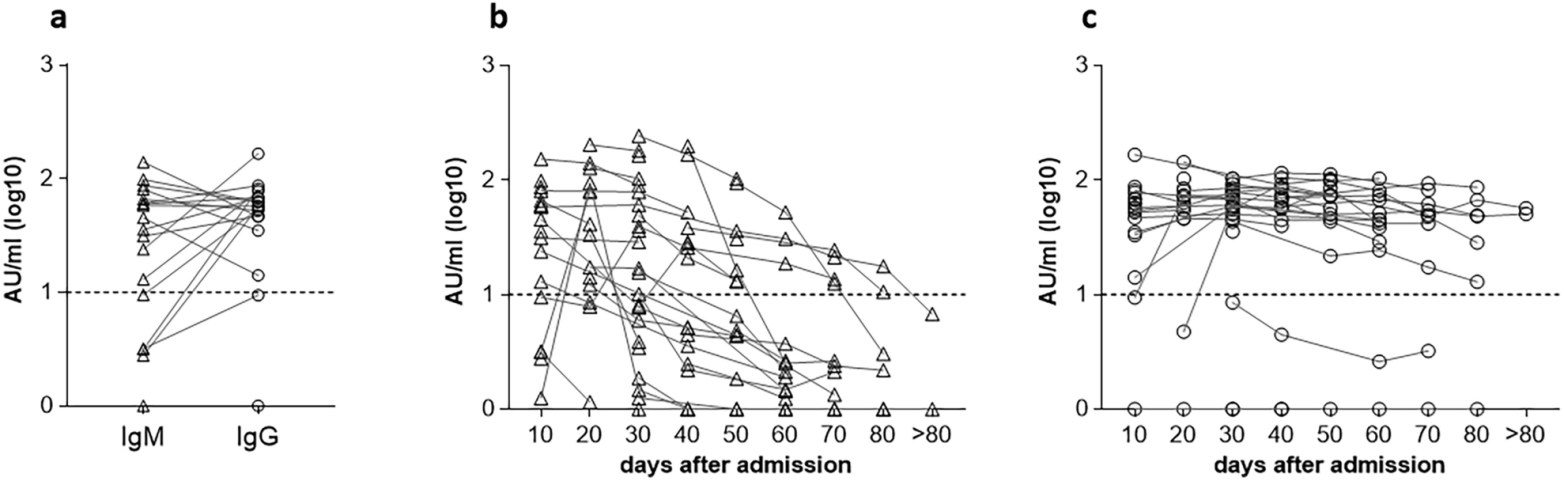
Anti-SARS-CoV-2 IgM and IgG antibodies in serum. Serum samples were assayed by CLIA at the hospital. Anti-SARS-CoV-2 IgM and IgG levels expressed in log10 AU/ml of each patient are reported. The dotted lines indicate the cut off of the assay (10 AU/ml). (**a**) Anti-SARS-CoV-2 IgM and IgG levels in serum samples (N = 18) collected within 10 days from the hospital admission are shown. Kinetics of anti-SARS-CoV-2 IgM (**b**) and IgG (**c**) antibodies in all serum samples collected at different time points (days) after the admission.

A large variability of anti-SARS-CoV-2 IgM titers was observed in the sera collected within 10 days from the hospital admission, with 72.2% of patients showing anti-SARS-CoV-2 IgM levels over the threshold of the assay (median above the cut off: 59.8 AU/ml; 95% CI 31–87 ). At the same time, 88.9% of patients showed detectable levels of anti-SARS-CoV-2 IgG (median above the cut off: 59.9 AU/ml; 95% CI 47–77 ), indicating that they arrived at the hospital several days after primary infection when the Ig switch had already occurred (Fig. 1a). The kinetics of anti-SARS-CoV-2 IgM and IgG levels in patients from the first time point available are reported in Fig. 1b and 1c, respectively. Anti-SARS-CoV-2 IgG persisted up to the last examination in all patients, except for three of them showing always undetectable (N = 2) or slightly below the threshold levels (N = 1) throughout the hospitalization. As expected the virus-specific IgM response decreased over time. In particular, the % of patients with detectable IgM was 58.3, 23.1 and 0 at 30, 60 and > 80 days, respectively.

### Analysis of neutralizing antibodies in serum

Patient’s sera were assayed for the presence of neutralizing antibodies (nAbs) using a pseudovirus-based assay previously developed by us^11,14,15^. Here, this method was further validated in a subset of 19 sera, selected based on variable ID50 (negative, low, medium and high), after comparison of the ID50 values obtained using a live virus-based neutralization test (VNT).

**Fig. 2:**
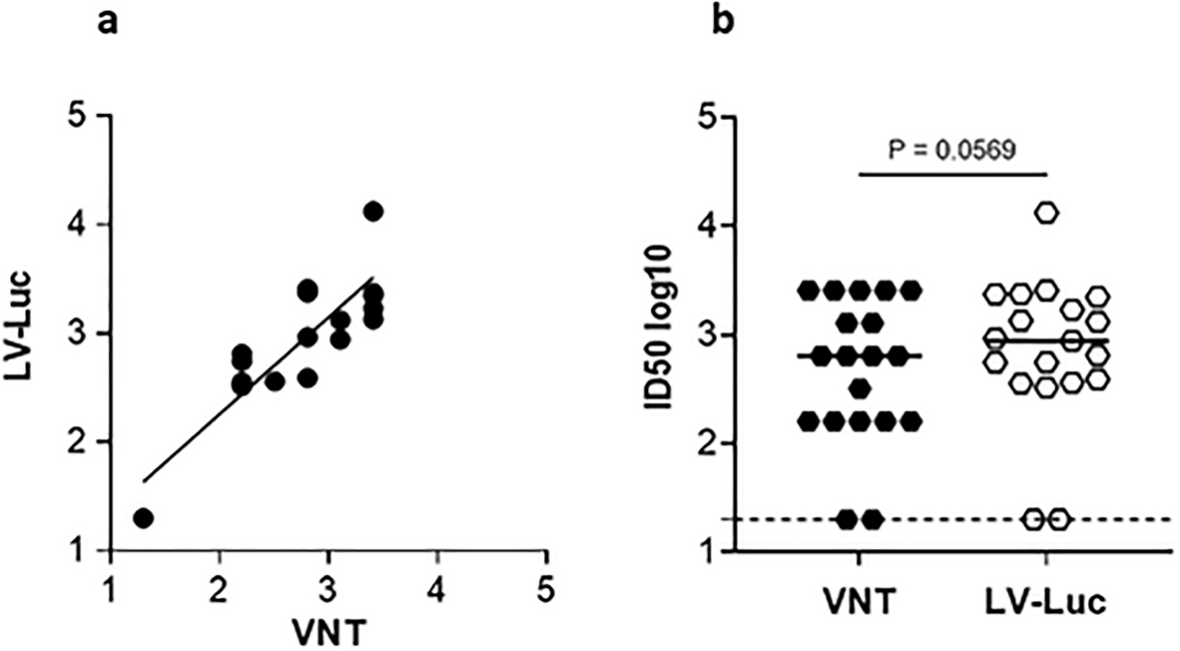
Comparison between neutralization assays. Selected serum samples (N = 19) were assayed using neutralization assays based on infectious SARS-CoV-2 (VNT) and on lentiviral vector pseudotyped with Spike (LV-Luc). (**a**) Correlation between the ID50 (log10) values obtained from the two assays. Dots correspond to individual measurements; the black line represents the regression line. (**b**) Comparison of ID50 (log10) values obtained using the two assays. A Wilcoxon matched-pairs signed rank test was used to compare the assays. The black line indicates the median ID50, the dotted line indicates the assay cut off (minimum serum dilution tested: 1:20 dilution).

Statistical analysis showed that the two neutralization assays correlated, as indicated by Spearman r coefficients (r = 0.811 *p* < 0.0001) (Fig. 2a). Altough the ID50 calculated using the pseudovirus assay were higher than those obtained using the VNT, the difference did not reach a significant p value (*p* = 0.0569) (Fig. 2b).

**Fig. 3:**
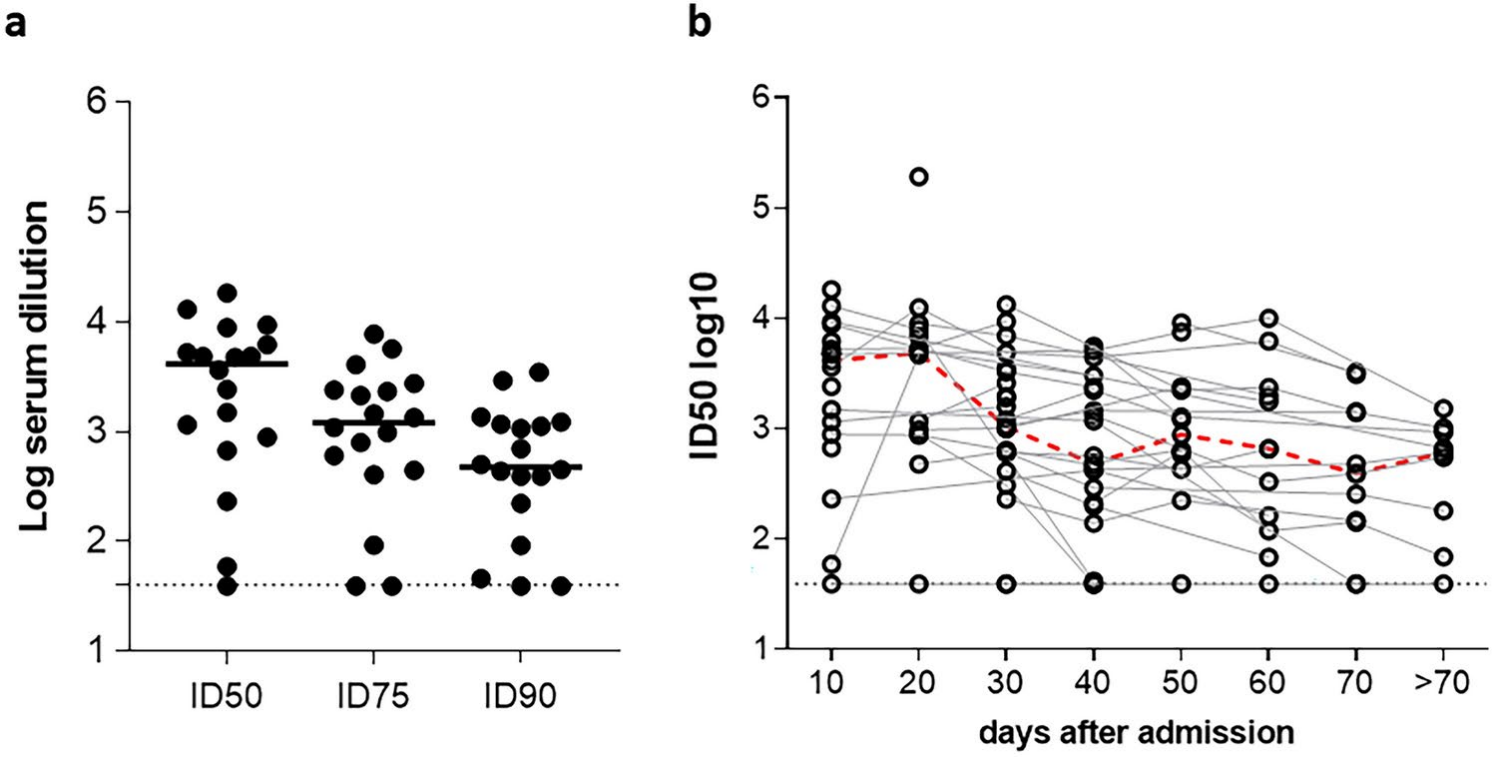
Analysis of anti-Wuhan nAbs in serum samples. (**a**) Serum samples collected within 10 days from the hospital admission (N = 18) were assayed by LV-Luc neutralization assay. Each dot represents a single patient. Data are expressed as ID50, ID75 and ID90. The black line indicates the median value. The dotted line indicates the assay cut off (minimum serum dilution tested: 1:40 dilution). (**b**) Kinetics of nAbs. Serum samples collected at different time points (days) after the admission were assayed for neutralization activity. Data are expressed as ID50. The red line represents the kinetics of median ID50.

The sera collected within 10 days from the hospital admission (N = 18) tested for the presence of nAbs directed towards the ancestral virus-derived Spike using the pseudovirus-based assay are shown in Fig. 3a. The majority of patients (17/18) showed neutralization activity in serum, measured as ID50 (median: 4166, 95% CI: 886–6185). ID75 and ID90 were calculated as well, showing median values of 1211 and 475, respectively (Fig. 3a). Although samples with undetectable nAbs were also negative for anti-SARS-CoV-2 IgG, no correlation was found between anti-SARS-CoV-2 IgG titers measured by CLIA and anti-Spike nAbs expressed as ID50 (r = 0.386, *p* = 0.216). The lack of correlation might be attributed to the presence of the Nucleoprotein as coating antigen in addition to Spike in the CLIA assay for the measurement of anti-SARS-CoV-2 IgG. The dynamic of nAb response showed a slight decrease over time but the neutralizing activity persisted in most of the patients (Fig. 3b).

### Analysis of anti-Spike IgG and IgA in nasopharyngeal swabs

Anti-Spike IgG and IgA were evaluated by ELISA in the available nasopharyngeal swabs (NPS) collected at different time points. Overall, anti-Spike IgA were detected in 30/32 NPS samples, while anti-Spike IgG were present in 28/30 NPS analyzed, collected at any time point.

**Fig. 4:**
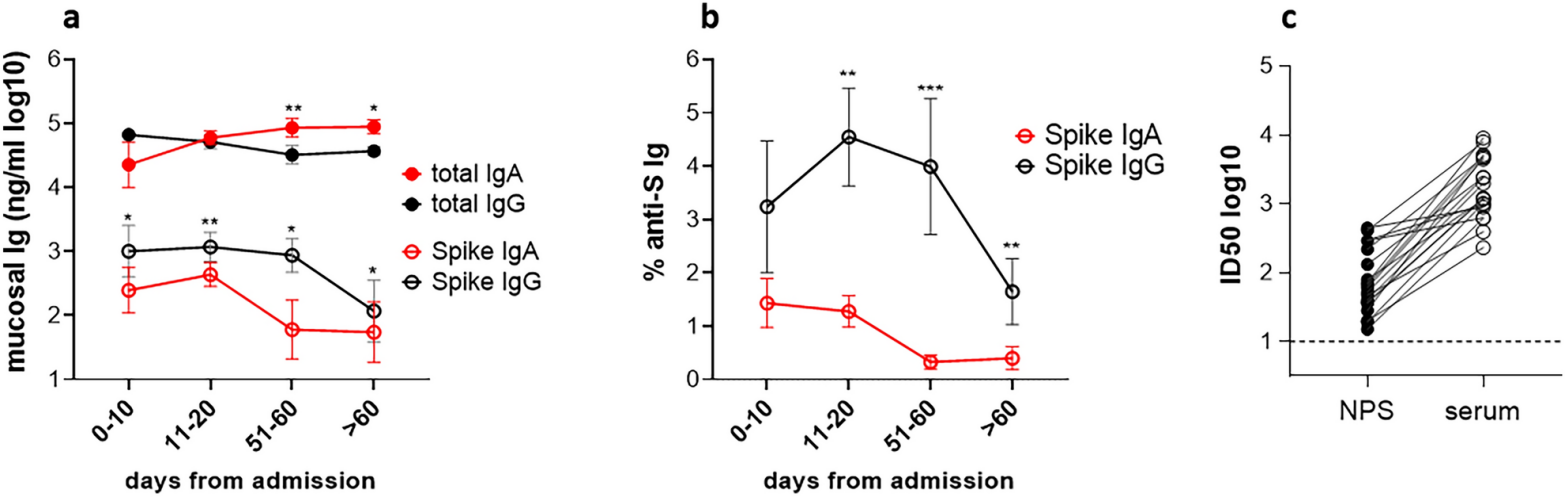
Kinetics of anti-Spike antibodies in NPS. Swabs collected at the hospital admission and during hospitalization were assayed by ELISA. (**a**) Kinetics of total IgG and IgA and anti-Spike (S) IgG and IgA (mean ± SEM). Data are expressed as ng/ml. (**b**) Kinetics of the percentage of anti-Spike IgG and IgA. Asterisks indicate a significant difference between IgG and IgA (*p < 0,05, **p < 0,01, ***p < 0,001, Wilcoxon matched-pairs signed rank test). (**c**) Evaluation of neutralizing activity in NPS. Selected patients (N = 19) with serum nAbs (ID50 > 100) were assayed for anti-Wuhan nAbs in NPS. The corresponding value in serum is also indicated. Each dot represents a single patient. Results are expressed as ID50. The dotted line indicates the assay cut off (minimum sample dilution tested: 1:10 dilution).

Due to the inter-individual variability and the variability of sampling, total IgA and total IgG in NPS were also measured. While total IgA and IgG persisted over time, both Spike-specific IgA and IgG decreased in terms of concentration (Fig. 4a) and more evidently in terms of % (specific Ig/total Ig × 100) (Fig. 4b). Data showed that concentration and % of anti-Spike IgG levels in NPS were significantly higher compared to anti-Spike IgA at any time point.

### Analysis of neutralizing Ab in nasopharyngeal swabs

Samples of selected patients (N = 19) with detectable levels of serum nAbs (ID50 > 100) were assayed for presence of anti-Wuhan-Hu-1 Spike nAbs in NPS. All patients showed nAb activity in NPS, although at levels lower than those observed in sera (median ID50: 60 and 1205 with 95% CI: 52–296 and 886–4715, in NPS and serum, respectively), which is, at least in part, likely due to the dilution of the samples during swab collection** (**Fig. 4c). Although the NPS samples showed neutralization activity, we did not observed a significant correlation between ID50 in NPS and sera (r = 0.2474 *P* = 0.3072). Again, this result could be attributed to sampling procedures, i.e. the different amount of sample collected by swabs.

### Quantification of SARS-CoV-2 RNA in nasopharyngeal swabs

**Fig. 5:**
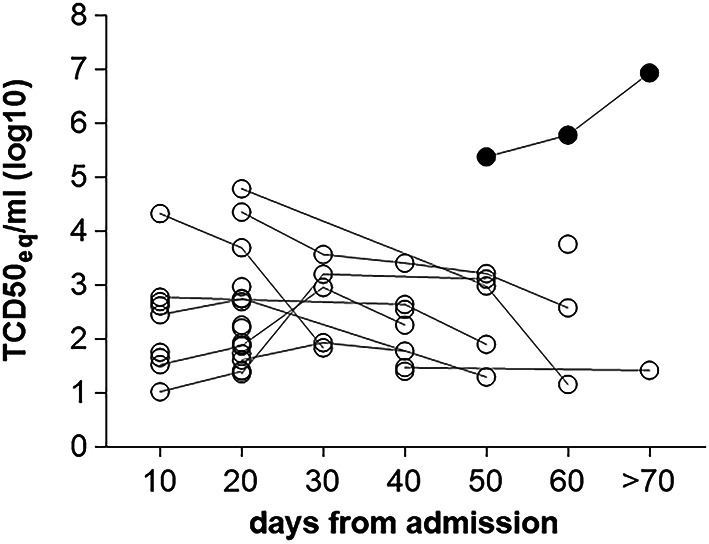
Analysis of viral RNA content in NPS. Quantification of viral RNA in the available NPS by RT-PCR at different time points from the hospital admission (N = 28). Data are expressed as TCD50_eq_/ml. Black circles indicate RNA content of a patient with a peculiar immune profile discussed in the text.

Quantification of the virus content in NPS collected at different time points after admission was performed by qRT-PCR. The viral load in samples collected within 10 days from the hospital admission (N = 10) ranged from 0.11 × 10^2^ and 5.14 × 10^4^ TCD50_eq_/ml. Although the viral load decreased over time, a remarkable persistence at late time points is shown (Fig. 5). Of note, one patient showed persistent viral load with over 10^5^ TCD50_eq_/ml at two months from the hospital admission (black circles in Fig. 5). The same patient did not mount any humoral responses nor neutralizing activity to SARS-CoV-2 in serum and in NPS at any time point analyzed starting from 30 days and up to 80 days after admission. The patient remained at the hospital for 111 days and recovered from the disease.

### Cross-neutralization activity in Sardinian patient’s sera and comparison with an analogous cohort of COVID-19-infected patients from a different region of Italy

**Fig. 6:**
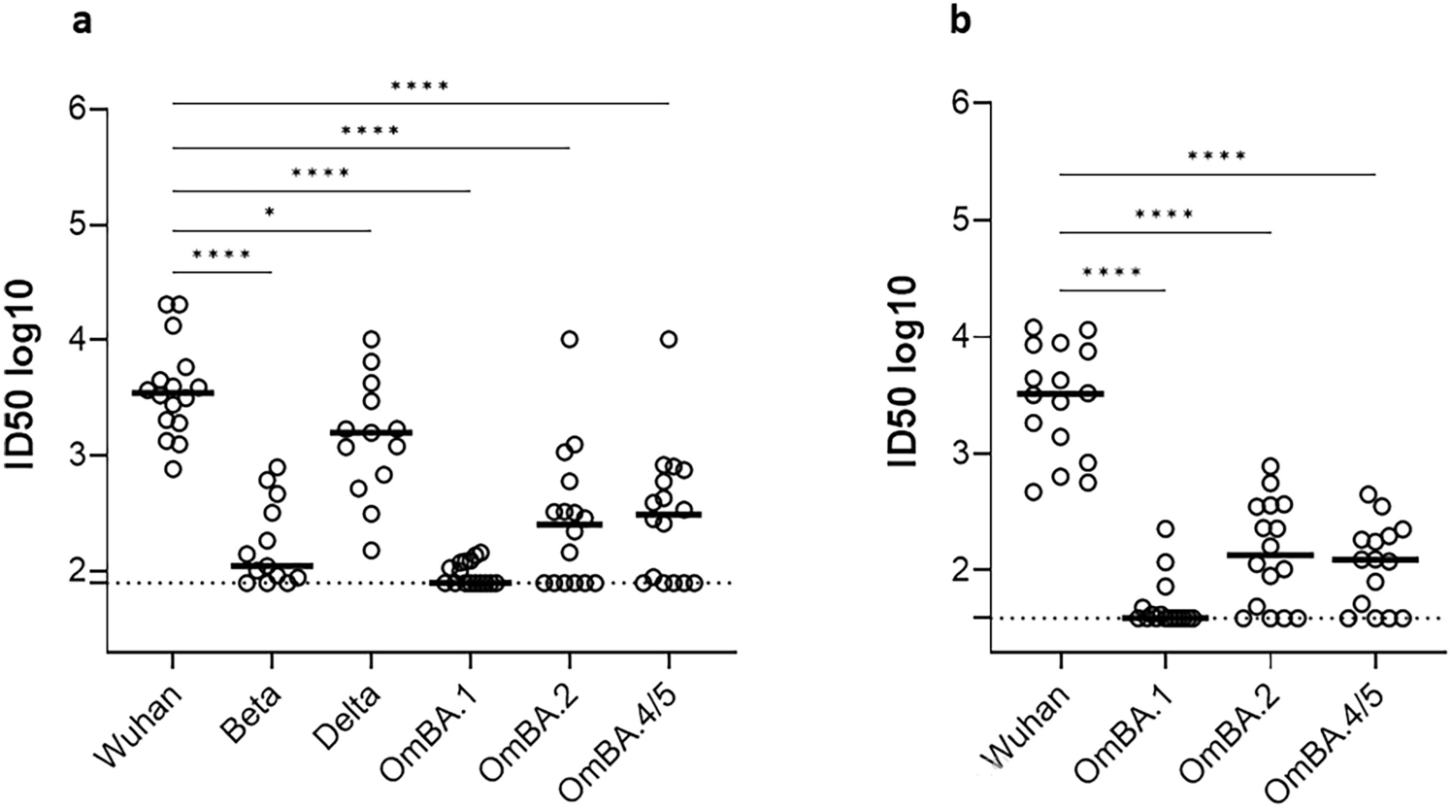
Cross neutralization with VoC. NAbs against each VoC were measured in serum samples (N = 16) from Cagliari (**a**) and Milan cohort (**b**), using LV-Luc pseudotyped with Spike from the indicated VoC. Results are expressed as ID50. Each dot represents a single patient. The black line indicates the median ID50. The dotted line indicates the assay cut off (minimum serum dilution tested: 1:80 or 1:40 starting dilution). The asterisks indicate a statistically significant difference between ID50 Wuhan versus ID50 VoC, measured by mixed-effects analysis (*p < 0,05; ****p < 0,0001).

The presence and the degree of cross-neutralization activity were measured in the serum of selected Sardinian patients (N = 16) with detectable nAbs against the Wuhan Spike (ID50 > 300, median: 3516 with 95% CI: 1918–5859, Table 2). The serum of patients showed a significantly lower neutralizing activity against all tested VoC compared to the parental virus. In particular, a dramatic reduction of neutralization activity was observed against Beta (median: 141, 95% CI: 88–617) and Omicron VoC (OmBA1: < 80, 95% CI: 79–122; OmBA.2: 255, 95% CI: 79–602 and OmBA.4/5: 294, 95% CI: 79–599). The cross-reactivity against Delta (median ID50: 1645, 95% CI: 521–6526) was partially maintained, although still significantly lower (Fig. 6a).

**Table 2:** Characteristics of the San Raffaele (OSR) and SS.Trinità Hospital COVID-19 study population (N = 16) selected for the evaluation of cross-neutralization activity.

Characteristics (N, %)	OSR	SS. Trinità
Age, years (median, 95%CI)	56,5 (47–68)	66 (44–81)
Anti-Wuhan nAbs ID50 (median, 95% CI)	3250 (841–8613)	3516 (1918–5859)
Sex Male	11 (68.7%)	10 (62,5%)
Ethnicity		
Caucasian	16 (100%)	16 (100%)
Co-morbidities		
Hypertension	8 (50%)	7 (43,7%)
Obesity	3 (18.7%)	1 (6.2%)
Coronary Artery Diseases	4 (25%)	4 (25%)
Rheumatoid arthritis	0	1 (6.2%)
Cancer	1 (6.2%)	1 (6.2%)
Median days of hospital stay (95%CI)	14 (6–25)	35 (23–69)
ICU	4	4

ICU: intensive care unit.

To verify whether the observed Omicron BA.1 escape was a peculiar characteristic of Sardinian patients, the cross-neutralizing activity against all the Omicron VoC was evaluated in an analogous cohort of patients from different geographical origins collected in Lombardy at San Raffaele Hospital (OSR, Milan, Italy) (Table 2). As shown in Fig. 6b, the results obtained from samples in this cohort paralleled the results obtained with patients from Cagliari, showing a strong and significant reduction of ID50 against the Omicron subvariants, particularly BA.1 (ID50 median Wuhan, BA.1, BA.2 and BA.4/5: 3520, 95% CI: 841–8613; < 40, 95% CI: 39–48; 137, 95% CI: 39–361 and 122, 95% CI: 39–197, respectively).

## Discussion

Although the COVID-19 pandemic is now under control in terms of hospitalizations and deaths, it is still important to retrospectively evaluate the immune response after infection with emerging SARS-CoV-2 in patients from different geographical locations and genetic backgrounds, in preparation of new and/or re-emerging infectious disease pandemics. Sardinian population represents an interesting paradigm due to peculiar immunogenetic characteristics that may confer protection against severe COVID-19 and may help to explain a lower fatality rate during the pandemic^18^ and a lower infection rate and incidence of hospital admissions for severe COVID-19 related to SARS-CoV-2 compared to the other Italian districts^2,6^.

In this study we retrospectively profiled the immune response of symptomatic COVID-19 patients from Cagliari, Sardinia, during the first wave of the pandemic by using additional assays to those available during the first wave of the pandemic, when diagnostic tools were limited. Also, we demonstrated the importance of collaborative interactions between hospitals and research laboratories. The evaluation of humoral response was possible using methodologies developed in our laboratory, which were either validated in previous studies, including the neutralization assay based on lentiviral vectors pseudotyped with ancestral SARS-CoV-2 Spike and VoC^14,15,19–21^ and quantification of viral RNA in NPS^22^ or set up in this study, including the ELISA assay for the measurement of anti-Spike IgG and IgA in NPS.

Anti-SARS-CoV-2 IgM were detectable early in 72% of patients and decreased over time, starting from 30 days after the admission. Most patients (44 out of 46) showed presence of anti-SARS-CoV-2 IgG at admission, which persisted up to the last examination (80 days). This behavior is consistent with an expected immune response to viral infection, showing various scenarios of IgG and IgM association, mostly depending on the onset of infection and the timing of the immunoglobulin class switch in the patients^23–26^. Similarly to IgG, patients showed presence of neutralizing activity against SARS-CoV-2 using a pseudovirus-based assay, which persisted as well up to the last examination. The severity of infection accounted for persistent humoral responses that we observed up to 4 months from hospital admission, consistent with previous findings evaluating anti-S Abs in severe cases^27,28^, and analyzed up to 1 year from infection^25,29^.

Selected patients with higher levels of neutralizing activity against ancestral Wuhan-Hu-1 Spike were also evaluated for the presence of cross-neutralizing activity against later VoC. Results indicated that patients maintained a cross-neutralizing activity against Delta VoC only, while cross-nAbs against Beta and Omicron VoC showed different degrees of reduction. In particular, we found a remarkable reduction of nAbs against BA.1 compared to BA.2 and BA.4/5 Omicron subvariants.

The lower neutralizing reactivity against BA.1 was unexpected and in contrast with studies in human subjects vaccinated with Wuhan-based vaccines and/or infected with different variants^30,31^, and with preclinical studies using different vaccine platforms delivering ancestral Spike sequences^14,32^, that reported a higher reduction in nAbs against BA.4/5 compared to other Omicron variants, including BA.1.

To verify whether the different neutralizing activity against the Omicron subvariants in our patient population from Sardinia might be attributed to the peculiar genetic background, we measured nAbs against BA.1, BA.2 and BA.4/5 Omicron subvariants using convalescent sera collected from an analogous cohort of patients hospitalized during the first wave of the pandemic at the San Raffaele hospital in Milan, Lombardy, a different geographical location. The results obtained from this group of patients confirmed the same pattern in the degree of neutralizing activity against the Omicron subvariants, showing the maximal reduction in reactivity against BA.1 compared to BA.2 and BA.4/5. Interestingly, this is in agreement with recent studies using convalescent sera from individuals infected during the first wave with the ancestral virus variant^9,10^ and underlines the difference between natural infection and vaccination in the induction of cross-reactive nAbs against VoC, suggesting that the BA.2 and BA.4/5 omicron variants are placed antigenically between pre-omicron variants and the BA.1 omicron variant, thus highlighting the importance of testing nAb activity in sera of both vaccinated and naturally infected individuals.

Since the respiratory mucosa is the port of entry and first line of defense against respiratory viruses such as SARS-CoV-2, mucosal immunity is of great importance for the prevention of viral replication and spread^33,34^. The longitudinal trend of anti-SARS-CoV-2 antibody response in NPS showed that both anti-Spike IgA and IgG were present in 98% of patients with available samples and remained detectable in most of them throughout the study, confirming earlier results^35,36^. Although we did not investigate the origin of the anti-Spike IgA and IgG detected in NPS, we provide evidence that the concentration of anti-Spike IgG in NPS was always significantly higher compared to anti-Spike IgA. This is in agreement with previous observations^37^, and suggests that anti-Spike Ig detected in NPS may derive from a pool of Ig both locally produced and transudated from serum. Importantly, anti-Spike Ig from NPS showed neutralizing activity. The lack of correlation between the level of nAbs in serum and NPS may be a consequence of the large variability in NPS specimen collection, also due to the invasiveness of the procedure, that may produce technical bias in obtaining clinical samples of adequate quality and similar amount, as discussed elsewhere^38^.

Viral replication was measured in NPS by qRT-PCR. Results showed that the virus was remarkably persistent in this patient population. Although PCR positivity starts to decline by week 3, the interpretation of this result may only indicate the detection of fragments of viral RNA and may not necessarily be associated to the presence of replication competent virus^39^. Our results are in line with data from literature, showing high variability of viral load regardless the virus variant, the vaccination and the time after the infection^40,41^.

Although the aim of the present study was focused on the humoral response to SARS-CoV-2, the mortality rate that we observed was 13.0%, compared to a range from 26 to 46.0% found in clinical studies from larger cohorts of patients from North Italy hospitals during the same timeframe^42,43^. From the beginning of the COVID-19 pandemic, the different rates of morbidity and mortality, ranging from 16 to 78%^44–46^, have been associated with ethnically different population^47–49^. Although we could not evaluate the HLA characteristics of the patients, it has been shown that host genetic factors are associated with SARS-CoV-2 infection and severity, and that the expression of specific HLA alleles could influence susceptibility to SARS-CoV-2 and severity of COVID-19^3–7,50–52^. Further studies will be necessary to investigate this aspects in more details, including the correlation with the anti-SARS-CoV-2 immune response.

This retrospective study has some limitations, primarily due to the limited number of patients, which reflects the low rate of hospitalizations that occurred in Sardinia during the first wave of the pandemic. Based on information reported at the Istituto Superiore di Sanità, which acts as a central Italian repository of case reporting, the total number COVID-19 reported hospitalization in the province of Cagliari during the first wave of pandemic from February to May 2020 was 54^53,54^. Due to the small number of individuals overall hospitalized in Cagliari and to the incomplete availability of samples, we could not evaluate differences with respect to age and/or gender or the association with other concomitant diseases, which may have uncovered differences within the cohort. Also, we did not investigate the HLA or other genetic characteristics of the patients, which limits the possibility to correlate the results to some genetic trait peculiar to individuals of Sardinian origin. While the cohort size is relatively small, one of the strengths of this retrospective study is the similar severity of COVID-19 infection among all patients who were of the same geographical origin.

In conclusion, this retrospective study gave us the unique possibility to profile the immune response induced by the ancestral Wuhan-Hu-1 SARS-CoV-2 in a vaccine-naïve population of patients from Cagliari, Sardinia. Although we did not observed a peculiar antibody immune response in this cohort, compared to other published studies^23–26^, and to a similar cohort that we analyzed here, the evaluation of the antigenic characteristics of ancestral Wuhan-Hu-1 SARS-CoV-2 in an infected population, naïve to previous infections, may help to better understand neutralization escape after infection with evolved viral strains, including VoC, or after vaccinations. Since the neutralizing activity against early Omicron VoC was reduced, compared to earlier VoC, further profiling of the immune response against later VoC, including more recent BA and XBB strains, will be of great importance to provide new additional tools helping the health system for pandemic preparedness, readiness and prompt response.

## Supplementary Information

Supplementary Information.

## Data Availability

The raw data supporting the conclusions of this article will be made available upon request by the corresponding authors, Andrea Cara and Donatella Negri.
