# Supplementary material for: Viral and immune profiles during the first wave of SARS-CoV-2 infection in hospitalized patients in Sardinia, Italy
Source: Sci Rep. 2025 Feb 24;15:6660. doi: 10.1038/s41598-025-90324-5 (PMC11850715; doi:10.1038/s41598-025-90324-5)
Supplement: Supplementary file 1 — Supplementary Information. [file 41598_2025_90324_MOESM1_ESM.docx]

**Supplementary Table 1**: laboratory blood tests at SS Trinità hospital (N=46)

|  |  | **All** | | **F** | | **M** | |
| --- | --- | --- | --- | --- | --- | --- | --- |
|  |  | N=46 | | N=15 (32,6%) | | N=31 (67,4%) | |
|  |  | median | 95%CI | median | 95%CI | median | 95%CI |
| WBC | x10^9^/L | 7,05 | 5,9-8,3 | 7,30 | 5,1-10,2 | 6,70 | 5,9-8,3 |
| Ly | x10^9^/L | 1,00 | 0,8-1,1 | 1,00 | 0,9-1,5 | 0,90 | 0,7-1,2 |
| N | x10^9^/L | 5,35 | 4,3-6,2 | 5,40 | 2,1-6,2 | 5,30 | 4,5-6,8 |
| Mo | x10^9^/L | 0,50 | 0,4-0,6 | 0,50 | 0,2-0,6 | 0,50 | 0,4-0,6 |
| N/Ly | ratio | 5,45 | 3,8-7,2 | 4,13 | 1,9-5,7 | 6,50 | 4,4-9 |
| PLT | x10^9^/L | 194,00 | 172-237 | 232,00 | 177-335 | 183,00 | 144-220 |
| Hb | gr/dL | 13,15 | 11,8-14,2 | 11,70 | 10,4-12,2 | 14,20 | 13,1-14,7 |
| T Bil | mg/dL | 0,53 | 0,53-0,91 | 0,56 | 0,48-0,9 | 0,62 | 0,5-0.9 |
| AST | U/L | 33,00 | 29-43 | 23,00 | 19-45 | 38,50 | 30-58 |
| ALT | U/L | 27,00 | 21-38 | 21,00 | 16-34 | 31,00 | 23-45 |
| Cr | mg/dL | 0,83 | 0,78-0,9 | 0,76 | 0,64-0,8 | 0,88 | 0,8-0,93 |
| LDH | U/L | 287,50 | 246-325 | 245,00 | 216-305 | 306,00 | 264-363 |
| CRP | mg/L | 95,15 | 51,8-125,8 | 52,71 | 26,7-136,8 | 100,25 | 59,6-137 |
| D-Dimer | µg/mL | 1,50 | 0,94-2,16 | 1,09 | 0,4-2,77 | 1,85 | 0,9-3.3 |
| Ferritin | ng/mL | 1031,44 | 632-2271 | 268,32 | 162-2271 | 1796,62 | 797-2938 |

F: female; M: male.

WBC: white blood cells; N: neutrophils; Ly: lymphocytes; Mo: monocytes; PLT: platelets; Hb: Haemoglobin; T Bil: bilirubin total; Cr: creatinine;
